# Gain-of-function PfAAT1 mutations compensate for impaired PfCRT function in *Plasmodium falciparum*

**DOI:** 10.21203/rs.3.rs-9887667/v1

**Published:** 2026-06-15

**Authors:** Muhammad M. Hasan, Eva S. Istvan, Jack Farah, Kharizta Wiradiputri, Sydney Gavula, Elizabeth A. Winzeler, Manuel Llinás, David A. Fidock, Daniel E. Goldberg

**Affiliations:** 1 Division of Infectious Diseases, Washington University School of Medicine, Saint Louis, MO USA; 2 Department of Microbiology & Immunology, Columbia University Irving Medical Center, NY, USA.; 3 Center for Malaria Therapeutics and Antimicrobial Resistance, Division of Infectious Diseases, Department of Medicine, Columbia University Irving Medical Center, NY, USA.; 4 Huck Center for Malaria Research (CmaR), The Pennsylvania State University, University Park, Pennsylvania, USA.; 5 Department of Chemistry, The Pennsylvania State University, University Park, Pennsylvania, USA.; 6 Department of Pediatrics, University of California, San Diego, La Jolla, CA USA.; 7 Skaggs School of Pharmacy and Pharmaceutical Sciences, University of California, San Diego, La Jolla, CA USA.; 8 Department of Biochemistry and Molecular Biology and Huck Center for Malaria Research, Pennsylvania State University, University Park, Pennsylvania, USA.; 9 Department of Molecular Microbiology, Washington University School of Medicine, Saint Louis, MO USA.

## Abstract

Mutations in the chloroquine resistance transporter (PfCRT) confer resistance to chloroquine (CQ) in the malaria parasite *P. falciparum*. Mutant PfCRT variants, defined by the K76T substitution, mediate efflux of CQ from the parasite digestive vacuole (DV), the site of drug action. However, these mutations impair the native function of PfCRT, which is export of hemoglobin (Hb)-derived peptides from the DV for downstream utilization as amino acids. Mutations in amino acid transporter 1 (PfAAT1) have also been associated with CQ resistance through population genomics and in vitro genetic crosses. The S258L mutation in Africa and the F313S mutation in Southeast Asia, often accompanied by additional substitutions, are found in near-complete linkage disequilibrium with *pfcrt* mutations, although the mechanistic basis linking these mutations to PfCRT dysfunction and CQ resistance has remained unclear. Here, we show that PfAAT1 is essential in parasites harboring mutant PfCRT, but largely dispensable in a wild-type PfCRT background. Replacing the mutant *pfcrt* allele with the wild-type version in otherwise isogenic parasite lines abolished the growth defect associated with *pfaat*1 knockdown, indicating functional overlap between PfCRT and PfAAT1. Consistent with this, PfAAT1 knockdown results in accumulation of Hb-derived peptides. Field-derived PfAAT1 variants (S258L and F313S) confer a significant fitness advantage over the wild-type allele in the mutant PfCRT background, consistent with a gain-of-function that enhances compensation for impaired PfCRT peptide transport activity. Comparative growth analyses reveal that the F313S mutation imparts a pronounced fitness advantage over the wild-type PfAAT1 under cysteine- and methionine-limiting conditions, providing a functional basis for this gain-of-function phenotype. We find that PfAAT1 mutations do not independently alter CQ sensitivity. Our data suggest that *pfaat1* allele selection in the field is driven primarily by epistatic interaction with *pfcrt*.

## Introduction

The malaria parasite *Plasmodium falciparum* replicates within human red blood cells during the pathogenic stage of infection. Host cell hemoglobin (Hb) is internalized into the parasite digestive vacuole (DV), where globin polypeptides are degraded by DV proteases primarily into oligopeptides^1–3^. Excess free heme released during this process is highly toxic to the parasite and is converted into inert hemozoin crystals within the DV^4^. Hb-derived peptides are transported to the parasite cytoplasm, where they are further degraded into amino acids that are utilized for protein synthesis^5,6^.

Chloroquine (CQ), the first widely deployed synthetic antimalarial, exerts its activity by inhibiting hemozoin formation^7,8^. Resistance to CQ has arisen primarily through gain-of-function mutations in the chloroquine resistance transporter (PfCRT), a DV membrane protein that mediates CQ efflux from the vacuole^9–11^. The native function of PfCRT is to export Hb-derived peptides from DV to cytoplasm^12^. Notably, CQ-resistant PfCRT variants are impaired in this function, imposing a fitness cost in the absence of drug pressure^12–14^. Consistent with this, knockdown of PfCRT leads to accumulation of oligopeptides in the DV and a swollen DV phenotype, likely resulting from osmotic imbalances across the DV membrane^15^.

Recent studies have shown that the emergence of CQ-resistant *pfcrt* alleles in field isolates is frequently accompanied by mutations in amino acid transporter 1 (PfAAT1)^16,17^. Hereafter, ancestral alleles are referred to as wild type (WT) and variants as mutant alleles. While CQ-resistant *pfcrt* alleles typically contain multiple substitutions that vary across geographic regions, they are universally defined by the K76T mutation (*pfcrt*^K76T^)^18–20^. Mutant *pfaat1* alleles also show geographic variation, with the S258L variant predominating in Africa and F313S selected in Southeast Asia either alone or in combination with S258L, Q454E or K541N^17^.

PfAAT1 is a multipass transmembrane protein belonging to the amino acid/polyamine transporter 2 family. Among PDB structures, it is structurally most similar to sodium-coupled neutral amino acid transporter 9 from zebrafish^21^. Heterologous expression of PfAAT1 in yeast confers increased sensitivity to CQ, suggesting a potential role in CQ transport^22^. In *Plasmodium berghei*, the PfAAT1 homologue is dispensable during the asexual blood stage; however, knockdown of PbAAT1 results in a swollen DV phenotype accompanied by accumulation of specific Hb-derived peptides^23^.

Genetic crosses between the CQ-sensitive 3D7 strain (harboring *pfcrt*^WT^ and *pfaat1*^WT^ alleles) and the CQ-resistant NHP4026 strain (carrying *pfcrt*^K76T^ and *pfaat1*^S258L/F313S^ alleles) demonstrated strong selection for *pfcrt*^K76T^ under CQ pressure^17^, as expected. Strikingly, the mutant *pfaat1* allele was also highly enriched (98 out of 100 progeny clones). In the absence of CQ, *pfcrt*^WT^ was strongly favored in progeny clones, consistent with the fitness cost of mutant PfCRT. Notably, when *pfcrt*^K76T^ was retained without drug pressure, it predominantly co-occurred with *pfaat1*^S258L/F313S^, whereas the combination of mutant *pfcrt*^K76T^ with *pfaat1*^WT^ was significantly underrepresented, suggesting an epistatic interaction between these loci^17,24^. However, the mechanistic basis of this interaction and the functional role of PfAAT1 in the parasite remain unclear.

Here, we investigated the functional relationship between PfAAT1 and PfCRT by knocking down PfAAT1 in both CQ-sensitive and -resistant parasite backgrounds and assessing the effect of PfAAT1 mutations by complementing the knockdown lines with distinct *pfaat1* alleles. Coupled with metabolomics and media amino acid dropout experiments, the data define the functional role of PfAAT1 in amino acid acquisition in the parasite and explain its epistatic interaction with PfCRT, providing mechanistic insight into how compensatory mutations shape the evolution and fitness landscape of antimalarial drug resistance.

## Results

### PfAAT1 is a DV membrane protein that is essential in the CQ-resistant Dd2 strain.

**Figure 1.**
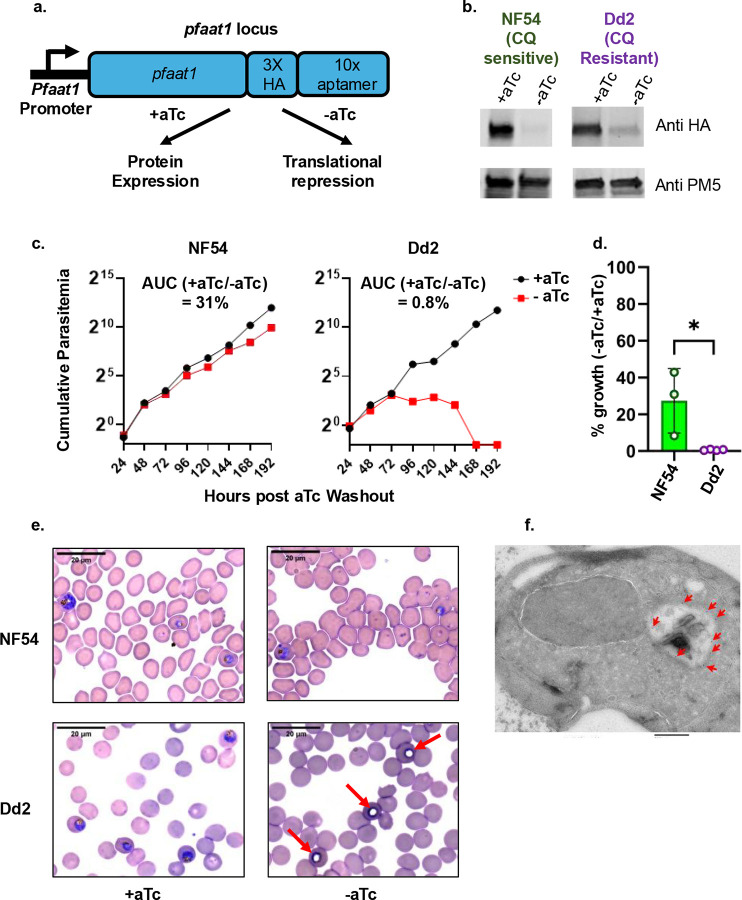
Conditional knockdown and localization of PfAAT1. a, Schematic of the *pfaat1* locus following CRISPR–Cas9–mediated editing to introduce a C-terminal 3×HA tag and a 10× aptamer cassette enabling anhydrotetracycline (aTc)-dependent translational control. b, Representative western blot showing PfAAT1 knockdown upon aTc washout. Samples were collected 48 h post–washout. Plasmepsin V (PM5) was used as a loading control. Data are representative of at least three independent experiments. Full blot images are provided in Supplementary fig. 2. c, Representative growth curves of PfAAT1 conditional knockdown (cKD) parasites cultured in the presence (+aTc) or absence (−aTc) of aTc. Parasitemia was measured by flow cytometry every 24 h for 8 days. Values represent cumulative parasitemia, corrected for dilution at each passage. Data represent mean ± s.d. from three technical replicates. Parasitemia is plotted on a log2 scale (zero parasitemia values are artificially plotted on the X axis); area under the curve (AUC) was calculated from linear-scale data. Percent AUC (−aTc/+aTc) is indicated. Each experiment was independently repeated 3–4 times. d, Quantification of growth phenotypes across strains using normalized AUC values from independent experiments. Data are shown as mean ± s.d. with individual data points representing independent experiments; statistical significance was determined by unpaired t-test (*P < 0.05). e, Hematological staining of parasites cultured in the presence or absence of aTc, 40 h post–washout. Red arrows indicate swollen, translucent digestive vacuoles (DVs) under PfAAT1 knockdown conditions. Scale Bars, 20 μm. f, Immunoelectron microscopy of GFP-tagged PfAAT1 in NF54 parasites. PfAAT1 was detected using anti-GFP primary antibody and 12 nm gold-conjugated secondary antibody. Red arrows indicate PfAAT1 localization at the DV membrane.

We tagged the endogenous PfAAT1 with a 3×HA tag together with a 10× aptamer that requires anhydrotetracycline (aTc) in the medium for translation^25^ (Fig. 1a, **Supplementary Fig. 1**). We used two lab-adapted *P. falciparum* strains for this purpose, both of which have attB sites integrated at the non-essential *cg6* locus for streamlined transgenic integration^26,27^. One is NF54, the parent of the reference *P. falciparum* strain 3D7, which encodes *pfcrt*^WT^ and *pfaat1*^WT^ alleles and is sensitive to CQ. The other is Dd2, a widely used CQ-resistant strain in which PfCRT differs from WT PfCRT at eight positions, including the K76T mutation (*pfcrt*^K76T^). Its *pfaat1* allele harbors the F313S mutation (*pfaat1*^F313S^).

ATc washout resulted in robust knockdown of PfAAT1 in both strains by the end of the first replication cycle (Fig. 1b, **Supplementary Fig. 2**). Knockdown of PfAAT1 significantly impaired the growth of NF54, but was not lethal (Fig. 1c,d). In contrast, PfAAT1 knockdown was lethal in the Dd2 strain, with parasites failing to replicate from the second cycle following aTc washout (Fig. 1c,d). Even during the first cycle, hematological staining of Dd2 parasites 40 h post–aTc washout revealed a swollen, translucent DV in the knockdown condition (Fig. 1e), reminiscent of the PfCRT knockdown phenotype^15^. We followed parasite growth for up to 8 days (four replication cycles) and calculated the area under the curve (AUC) as a single summary measure of growth in each condition, normalizing the −aTc condition to the +aTc condition within each replicate (Fig. 1d).

Immunofluorescence microscopy using an anti-HA antibody revealed a predominantly circular staining pattern surrounding the hemozoin crystal (a distinctive dark region in the transmitted light channel), consistent with DV membrane localization (**Supplementary Fig. 3**). We also expressed a GFP-tagged PfAAT1 from the *cg6* locus in the NF54 strain (**Supplementary Fig. 4**). Immunoelectron microscopy on this line further confirmed localization of PfAAT1 to the DV membrane (Fig. 1f).

### PfAAT1 functionally complements PfCRT.

The swollen DV phenotype observed upon PfAAT1 knockdown in Dd2 resembles that of PfCRT knockdown^15^. In addition, the PfAAT1 knockdown is lethal in Dd2. Based on these observations, we hypothesized that PfAAT1 functionally complements the peptide transport/amino acid acquisition defect in *pfcrt* ^K76T^ CQ-resistant parasites such as Dd2.

**Figure 2.**
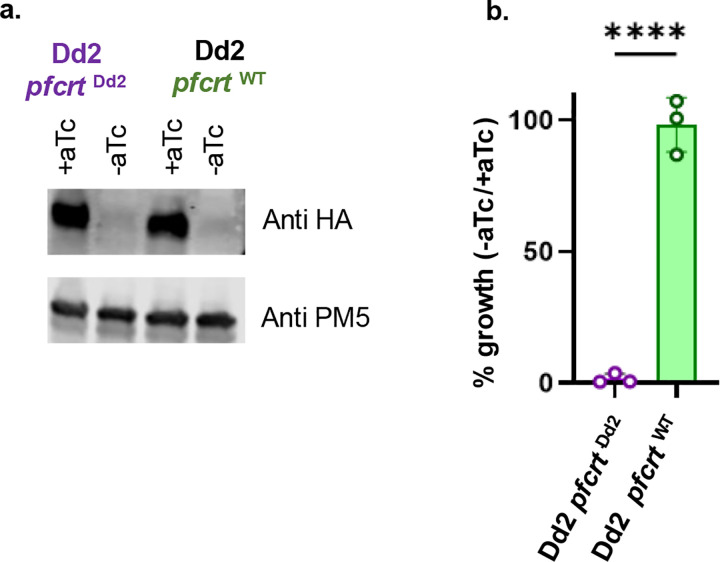
PfAAT1 knockdown in an isogenic Dd2 parasite pair expressing mutant or T PfCRT. a, Representative western blot showing PfAAT1 conditional knockdown upon aTc washout in an isogenic Dd2 parasite pair differing only at the *pfcrt* locus (*pfcrt*^Dd2^ vs *pfcrt* ^WT^). Samples were collected 48 h post aTc washout. Plasmepsin V (PM5) was used as a loading control. Data are representative of two independent experiments. Full blot images are provided in Supplementary Fig. 5. b, Quantification of growth phenotypes across the isogenic pair using normalized area under the curve (AUC) values from independent experiments. Data are shown as mean ± s.d. with individual data points representing independent experiments; statistical significance was determined by unpaired t-test (****P < 0.0001).

Because NF54 and Dd2 differ at numerous loci beyond *pfcrt*, we tested this hypothesis using an isogenic parasite pair differing only at the PfCRT sequence. This pair was generated by first deleting all *pfcrt* intron sequences in Dd2 to create an isogenic control (Dd2 *pfcrt*^Dd2^) and subsequently replacing this allele with the WT PfCRT sequence (Dd2 *pfcrt*^WT^)^28,29^. PfAAT1 was conditionally knocked down in both lines (Fig. 2a, **Supplementary Fig. 5**). Knockdown of *pfaat1* was lethal in the isogenic control, consistent with the parental Dd2 strain, whereas it had no detectable effect on the growth of Dd2 parasites expressing WT PfCRT (Fig. 2b). This complete rescue of the lethal growth defect of PfAAT1 knockdown in Dd2 by swapping the dysfunctional mutant PfCRT with WT PfCRT supports our hypothesis that PfAAT1 and PfCRT have partially redundant or overlapping functions.

### PfAAT1 knockdown leads to accumulation of Hb-derived peptides.

**Figure 3.**
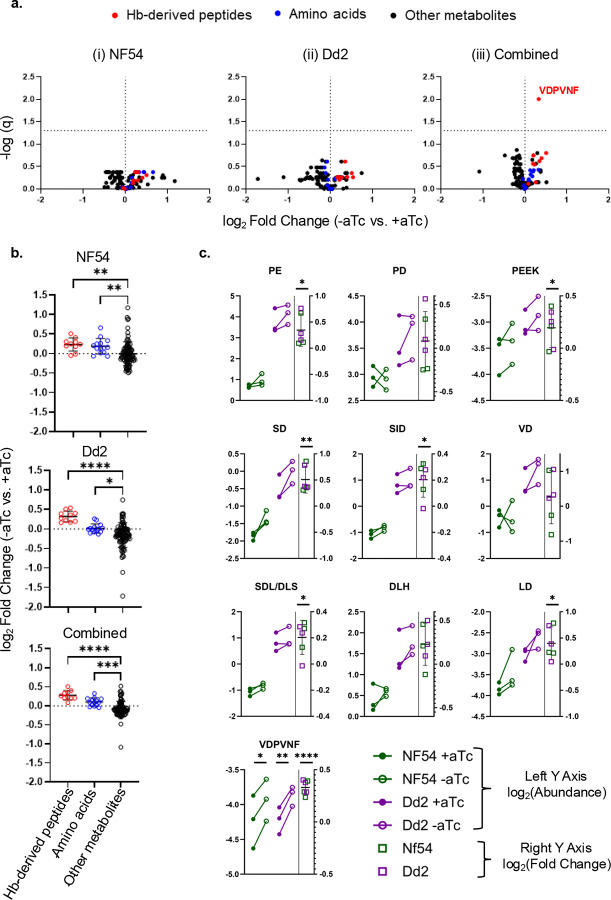
Quantitative metabolomics of F54 and Dd2 parasites with or without PfAAT1 knockdown. a, Volcano plots showing differential enrichment of detected metabolites upon PfAAT1 knockdown in (i) NF54, (ii) Dd2 and (iii) combined analyses. The x axis represents log_2_ fold change (−aTc versus +aTc), and the y axis represents −log_10_(q), where q values were calculated using a two-stage step-up false discovery rate (FDR) correction (Benjamini, Krieger and Yekutieli) from paired analyses. Dotted lines indicate log_2_ fold change = 0 and q = 0.05. Each strain includes three paired samples; the combined analysis includes six paired samples. Hb-derived peptides are shown in red, amino acids in blue and all other metabolites in black. The only significantly increased metabolite in the combined analysis is labeled. b, Differential enrichment of Hb-derived peptides, amino acids and all other detected metabolites grouped under these classifications in NF54, Dd2 and combined analyses. Each point represents an individual metabolite; lines indicate mean ± s.d. Statistical comparisons were performed using a non-parametric Kruskal–Wallis test with Dunn’s multiple-comparison correction; only statistically significant pairwise comparisons are shown (*P < 0.05, **P < 0.01, ***P < 0.001, ****P < 0.0001). c, Abundance of individual Hb-derived oligopeptides (di- to hexa-peptides; sequences indicated as panel titles) in NF54 (green) and Dd2 (purple) parasites under +aTc and −aTc conditions. Left y axis shows log_2_-transformed, median-normalized abundance. Paired samples (n = 3 per strain) are connected by lines. Statistical significance within each strain was assessed using paired t-tests and is indicated above the paired comparisons. Right y axis shows log_2_ fold change (−aTc versus +aTc) for each paired sample (n = 6 total). Individual fold-change values are plotted alongside the mean ± 95% confidence interval. Statistical significance of the combined dataset was assessed using a paired t-test and is indicated above the fold-change values (*P < 0.05, **P < 0.01, ***P < 0.001, ****P < 0.0001). Only statistically significant differences are annotated.

To determine the functional consequences of PfAAT1 knockdown, we performed an untargeted metabolomics analysis. aTc was removed from NF54 and Dd2 PfAAT1 knockdown lines at the ring stage, and infected erythrocytes were harvested from both WT and knockdown conditions 32 h post–washout. A total of 119 metabolites were detected per run (**Supplementary File 1**). Differential enrichment analysis of paired samples did not identify any metabolites that were significantly altered between WT and knockdown conditions for either strain (Fig. 3a(i)(ii)).

However, combined analysis (paired −aTc vs +aTc across both strains) identified one significantly enriched metabolite under the PfAAT1 knockdown conditions, the Hb-derived hexapeptide VDPVNF (Fig. 3a (iii)).

This limited finding across the overall detected metabolome likely reflects the modest statistical power of the dataset (three paired replicates per strain) and the early time point analyzed (30 h post–aTc washout). Although this time point preceded the onset of pronounced growth defects, it enabled stage-matched comparisons that would have been difficult to achieve at later time points. We therefore focused our subsequent analyses on specific metabolite classes. Given the functional overlap between PfAAT1 and PfCRT, we examined Hb-derived peptides in greater detail (Fig. 3b).

We identified 10 putative Hb-derived oligopeptides ranging from 2 to 6 residues in length^30–32^. As a class, these peptides were modestly (17% in NF54 and 25% in Dd2) but statistically significantly increased in both strains upon PfAAT1 knockdown (Fig. 3b). At baseline, these peptides were more abundant in Dd2 than in NF54, and each peptide increased consistently upon PfAAT1 knockdown in Dd2 (Fig. 3c). Eight out of ten peptides exhibited the same trend in NF54, and seven of them reached statistical significance in the combined analysis (Fig. 3c). These results are consistent with a role for PfAAT1 in transporting Hb-derived peptides to the cytoplasm from the DV.

Because PfAAT1 belongs to the amino acid transporter superfamily, we also examined amino acid levels. Fourteen amino acids were confidently identified in the metabolite dataset. As a class, amino acids also showed a statistically significant increase although the magnitude of change (13% in NF54; 1% in Dd2) was smaller than that observed for Hb-derived peptides (Fig. 3b). However, none reached statistical significance individually in the combined analysis (**Supplementary Fig. 6**). Isoleucine, methionine, tryptophan and tyrosine were significantly increased upon PfAAT1 knockdown only in the NF54 strain. Interpretation of amino acid changes is less straightforward than that of Hb-derived peptides, as amino acids can originate from multiple cellular compartments in our samples. Nevertheless, these results raise the possibility that PfAAT1 may also contribute to amino acid transport.

### Common field mutations in PfAAT1 enhance fitness in a mutant PfCRT background.

**Figure 4.**
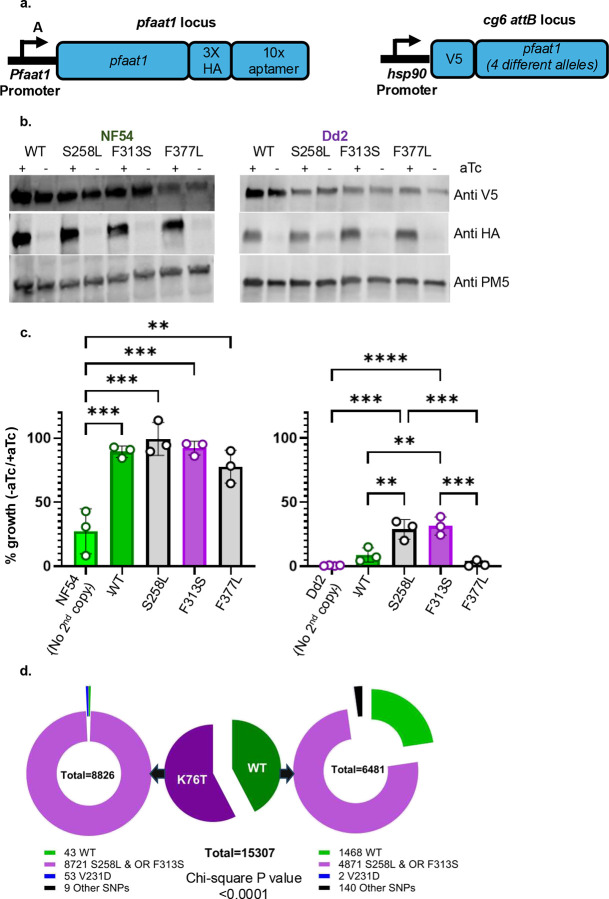
Complementation of PfAAT1 conditional knockdown with different alleles. a, Schematic representation of the complementation strategy, where a second copy of PfAAT1 is expressed from the *cg6 attB* locus. b, Western blots showing expression of both copies of PfAAT1 in both +aTc and −aTc conditions. Lanes are labeled with the identity of the V5-tagged PfAAT1 version expressed in each parasite line. Full blot images are provided in Supplementary Fig. 7. c, Quantification of growth rescue by complementation, normalizing AUC of −aTc condition by AUC of +aTc condition from growth curves over 8 days. Each point represents an independent experiment; bars show mean ± s.d. One-way ANOVA followed by Tukey’s multiple-comparison test was used to compare all groups within each strain. Only multiple-comparison–corrected significant differences are annotated with stars (*P < 0.05, **P < 0.01, ***P < 0.001, ****P < 0.0001). d, *pfaat1* and *pfcrt* genotypes of 15,307 *Plasmodium falciparum* isolates collected worldwide from 1966 to 2022. The middle pie chart divides isolates by the residue at position 76 of PfCRT, which are then subdivided by the indicated PfAAT1 variants on either side. Numbers of parasite isolates corresponding to each PfAAT1 variant are indicated in the labels. A chi-square test was performed to assess the association between PfCRT and PfAAT1 alleles. Only the chi-square *P* value is shown here; full statistical results are provided in Supplementary Fig. 9.

To assess the functional impact of field-derived PfAAT1 mutations, we complemented the PfAAT1 knockdown lines with a second copy of N-terminally V5-tagged PfAAT1 expressed from the *cg6* locus (Fig. 4a). In both NF54 and Dd2 backgrounds, we expressed four *pfaat1* alleles: the ancestral WT allele, the field-derived S258L and F313S variants (F313S is the natural variant in Dd2), and F377L, a mutation identified through in vitro selection with a compound unrelated to CQ and included as a non-field-derived control allele^33^ (Fig. 4b, **Supplementary Figs. 7, 8**). All alleles rescued the growth defect in NF54 (Fig. 4c).

In contrast, in the Dd2 background, the field-derived mutations (S258L and F313S) rescued the lethal growth defect significantly more effectively than either the WT allele or the laboratory-derived F377L variant (Fig. 4c). These results indicate that field-selected PfAAT1 variants confer a fitness advantage in the context of mutant PfCRT, consistent with a gain-of-function effect that functionally compensates for impaired PfCRT activity.

Although the field-derived variants rescued growth substantially better than WT PfAAT1, complementation remained incomplete in Dd2, including complementation with F313S, the endogenous *pfaat1* allele in this strain. The modest residual defect in rescue likely reflects limitations of our complementation system, such as altered expression from the heterologous *cg6* locus or effects of N-terminal epitope tagging, rather than an intrinsic functional defect of the F313S allele itself. This limitation was less apparent in NF54, where PfAAT1 knockdown is not lethal.

To assess which *pfcrt* and *pfaat1* alleles co-occur in field isolates, we consulted the *Plasmodium falciparum* Haplotype Atlas (Pf-HaploAtlas) database^34^, which stores haplotype information for all *P. falciparum* genes from 24,409 samples collected worldwide from 1966 to 2022. Among 15,307 samples with assigned alleles at both loci (**Supplementary file 2**), 98.81% of isolates harboring *pfcrt*^K76T^ also carried either or both *pfaat1*^F313S^ and *pfaat1*^S258L^ (Fig. 4d, **Supplementary Fig. 9**). This strong association (χ^2^ test, P < 0.0001) further corroborates our conclusion that these two mutations help PfAAT1 compensate for the functional defect in mutant PfCRT.

### The PfAAT1 F313S mutation enhances acquisition of methionine and cysteine.

**Figure 5.**
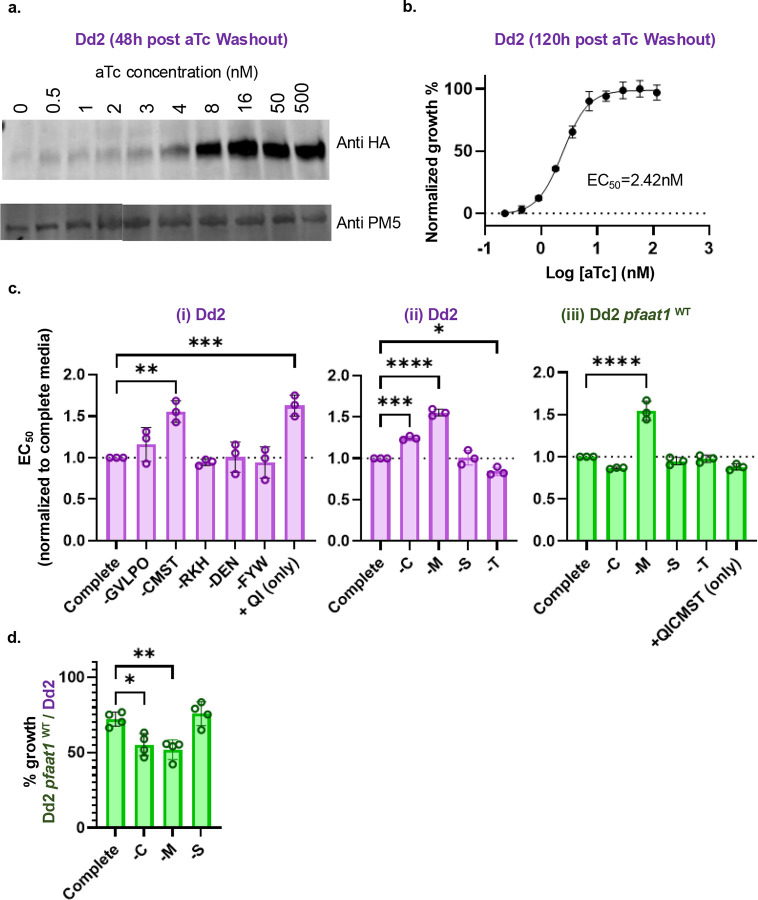
Amino acid dependency determination of different Dd2 PfAAT1 conditional knockdown strains. a, Western blot showing tuning of PfAAT1 expression by altering aTc concentration in the media. Samples were collected 48 h after aTc washout and readdition at specified concentrations. Anti-Plasmepsin V (PM5) was used as a loading control. Data are representative of two independent experiments. Full images of the blots are provided in Supplementary Fig. 10. b, A representative aTc dose–response curve of Dd2 *pfaat1* cKD parasites. Each point represents the mean of three technical replicates, and error bars show s.d. A four-parameter logistic model (log(agonist) versus response, variable slope) was fitted to normalized growth data. The calculated EC_50_ is indicated. c, aTc EC_50_ values determined in media lacking specific amino acids. (i) and (ii) were performed for parental Dd2 PfAAT1 conditional knockdown parasites (with F313S mutation of PfAAT1), while (iii) was performed for Dd2 parasites expressing WT PfAAT1 under translational control of aTc. Each data point represents EC_50_ values normalized to the EC_50_ determined for complete media in parallel experiments. One-way ANOVA followed by Bonferroni’s multiple-comparison test was used. In each panel, EC_50_ value were compared only with the complete media group; only Bonferroni-corrected significant differences are annotated (*P < 0.05, **P < 0.01, ***P < 0.001, ****P < 0.0001). d, Growth of Dd2 parasites expressing WT PfAAT1 from the endogenous locus, normalized to growth of Dd2 parasites expressing the native F313S allele, in different media conditions. Each point represents comparative fitness determined from parallel experiments performed under the same media condition. One-way ANOVA followed by Bonferroni’s multiple-comparison test was used to compare each amino acid–deficient condition to complete media; only Bonferroni-corrected significant differences are annotated (*P < 0.05, **P < 0.01, ***P < 0.001, ****P < 0.0001).

Hb-derived peptides are exported from the DV to the parasite cytoplasm, where they are further degraded into amino acids. We hypothesized that PfAAT1 contributes to the acquisition of specific amino acids derived from Hb digestion, and that field mutations enhance this activity. To test this, we exploited the tunability of the aptamer-based knockdown system, in which PfAAT1 expression can be modulated by varying aTc concentration (Fig. 5a, **Supplementary Fig. 10**). PfAAT1 levels yielding 50% normal growth can be quantified as an aTc EC_50_ in the Dd2 line (Fig 5b).

If acquisition of a given amino acid depends on PfAAT1, removal of that amino acid from the medium would require higher PfAAT1 expression to sustain growth, resulting in an increased aTc EC_50_. We first measured aTc EC_50_ in media lacking defined groups of amino acids (Fig. 5c (i)). Isoleucine and glutamine were not removed, as *P. falciparum* is auxotrophic for exogenous isoleucine^1,35,36^ and glutamine is needed for optimal growth, consistent with its central role in parasite metabolism^37^. As expected, EC_50_ increased in media lacking all tested amino acids. Notably, EC_50_ also increased in media lacking serine, threonine, cysteine and methionine. When tested individually, only methionine and cysteine deprivation significantly increased EC_50_ compared to complete media (Fig. 5c (ii)).

To assess allele-specific effects, we replaced the endogenous *pfaat1*^F313S^ allele in Dd2 with *pfaat1*^WT^ (**Supplementary Fig. 11**). In this background, methionine deprivation still increased EC_50_, whereas cysteine deprivation did not, indicating that both alleles support methionine acquisition, while the F313S mutation specifically enhances acquisition of cysteine or cysteine-containing peptides (Fig. 5c(iii)).

To test whether PfAAT1-dependent acquisition of methionine and cysteine underlies the fitness advantage of field mutations, we assessed the growth of Dd2 parasites expressing WT or F313S PfAAT1 in media lacking either methionine, cysteine or serine (as a control). Parasites expressing the WT allele exhibited reduced fitness compared to those expressing F313S under all conditions. Notably, this fitness difference was significantly more pronounced under cysteine- and methionine-depleted conditions relative to complete media (Fig. 5d).

Together, these results indicate that while both WT and F313S PfAAT1 support methionine acquisition, the F313S mutation confers a selective advantage under methionine limitation, likely through increased transport efficiency of methionine or methionine-containing peptides. In contrast, the allele-specific effect observed under cysteine limitation suggests a gain-of-function for cysteine or cysteine-containing peptide acquisition.

### PfAAT1 does not mediate CQ transport:

**Figure 6.**
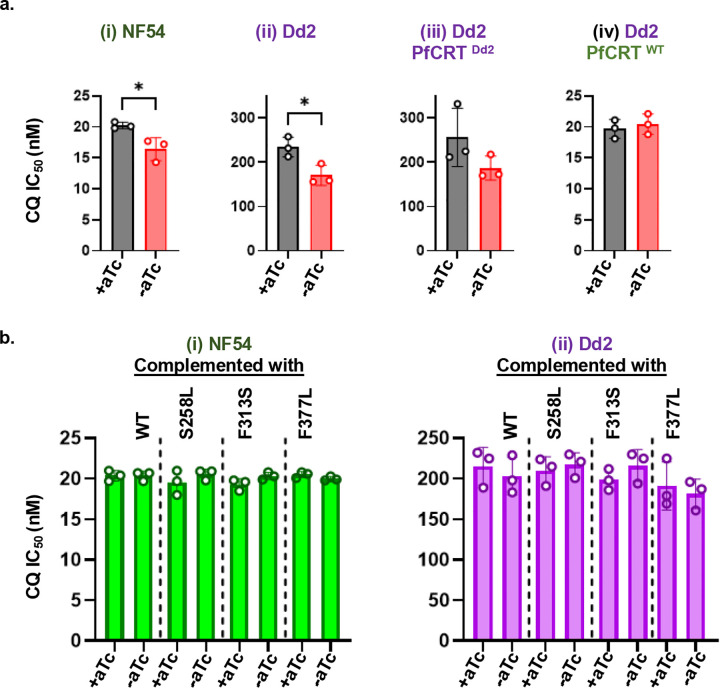
Chloroquine potency against PfAAT1 conditional knockdown parasites a, CQ IC_50_ values determined for PfAAT1 conditional knockdown parasites cultured in the presence (+aTc) or absence (−aTc) of aTc. b, CQ IC_50_ values determined for PfAAT1 conditional knockdown parasites complemented with different *pfaat1* alleles expressed from the *cg6* locus. Parasite strains are indicated above each panel. Bars represent mean ± s.d., and individual data points represent biological replicates. In a, statistical significance was determined using Student’s t-test between +aTc and −aTc conditions. In b, statistical comparisons within each panel were performed using ordinary one-way ANOVA followed by Bonferroni’s multiple-comparison test. Only significant differences are annotated (*P < 0.05, **P < 0.01, ***P < 0.001, ****P < 0.0001).

Given the functional overlap between PfAAT1 and PfCRT in peptide transport, we asked whether PfAAT1 also contributes to CQ efflux from the DV. We first measured CQ EC_50_ under WT and PfAAT1 knockdown conditions in both Dd2 and NF54 strains and observed a small but statistically significant decrease in EC_50_ upon knockdown (Fig. 6a (i and ii)). This initially suggested that PfAAT1 might contribute to CQ transport.

However, as PfAAT1 knockdown impairs parasite growth, we considered the possibility that reduced growth rate could confound CQ EC_50_ measurements and artifactually increase CQ potency. To address this, we measured CQ EC_50_ following PfAAT1 knockdown in the isogenic Dd2 parasite pair differing only at the *pfcrt* locus (Fig. 2a and 2b). In the isogenic control line, where *pfaat1* knockdown is lethal, a CQ hypersensitivity trend was again observed (Fig. 6a(iii)). In contrast, in the *pfcrt* allele-swap line, where WT *pfcrt* restores growth despite robust PfAAT1 knockdown, CQ EC_50_ remained unchanged upon knockdown (Fig. 6a(iv)).

Expression of different *pfaat1* alleles from the *cg6* locus did not significantly alter CQ sensitivity, arguing against an allele-specific effect on CQ transport (Fig. 6b). Together, these results indicate that the apparent CQ hypersensitivity associated with PfAAT1 knockdown is secondary to growth defects and not due to a direct role in CQ transport.

## Discussion

The causal relationship between specific *pfcrt* mutations and CQ resistance has been firmly established through population genetics^38,39^, targeted genetic manipulation^9,10^, and direct in vitro functional assays^40,41^. In contrast, although mutations in *pfaat1* have been repeatedly associated with CQ resistance and shown to co-occur with specific *pfcrt* alleles, a clear functional explanation for this association has been lacking^16,17,42–47^. Here, we demonstrate that PfAAT1 and PfCRT have overlapping functional roles in the parasite. The first clue was that, similar to PfCRT, PfAAT1 localizes to the DV membrane, and its knockdown phenocopies the PfCRT knockdown^15^ by causing swelling of the DV in the Dd2 strain. The lethal growth defect observed upon PfAAT1 knockdown in CQ-resistant Dd2 parasites is fully rescued by replacing the mutant *pfcrt* allele, which is defective in peptide transport, with the ancestral WT *pfcrt* allele. This finding provides direct evidence that PfAAT1 compensates for the impaired native function of mutant PfCRT^12^.

In contrast to previous reports, we did not observe a direct effect of PfAAT1 mutations on CQ susceptibility in either CQ-sensitive or CQ-resistant parasites^17^. Instead, parasites expressing mutant PfAAT1 field variants displayed a significant fitness advantage over those expressing the WT allele in the absence of CQ. Together, these findings support a model in which common field mutations in *pfaat1* represent gain-of-function adaptations that mitigate the fitness cost associated with CQ-resistant *pfcrt* variants, rather than directly contributing to CQ transport.

Besides the S258L and F313S variants investigated here, our analysis of allele co-occurrence identified an additional *pfaat1* variant, V231D, that shows strong linkage disequilibrium with the *pfcrt* K76T mutation (Fig. 4d). Particularly enriched in South American isolates, this *pfaat1* mutation may represent another compensatory mutation selected in response to functional constraints imposed by mutant PfCRT.

Expression of PfAAT1 in yeast membranes hypersensitized cells to CQ, suggesting a possible role in CQ transport. However, this phenotype required high CQ concentrations (~1 mM) in the medium, raising questions about its physiological relevance in parasites^17,22^. Previous studies also reported that S258L and F313S modestly increased CQ resistance in already CQ-resistant parasites. However, comparison of the S258L/F313S double mutant with WT PfAAT1 produced a counterintuitive fitness result in which parasites expressing the WT allele were fitter in a mutant *pfcrt* background, despite co-selection of mutant *pfcrt* and *pfaat1* alleles in genetic crosses^17^. Although our experiments are not directly comparable because of differences in genetic background and the use of single rather than double *pfaat1* mutations, our data help explain the selection of mutant *pfaat1* alleles in a manner consistent with both genetic cross and population-level association data.

Because mutant PfCRT impairs the export of Hb-derived peptides^12^, which serve as a major source of amino acids for the parasite, compensatory mechanisms are likely to involve alternative routes for peptide or amino acid acquisition. Our data raise the possibility that peptides are the primary substrates transported by PfAAT1. Previously, it was shown that Hb is predominantly degraded into oligopeptides within the DV, and free amino acids are produced in negligible amounts within this compartment^2^. Consistent with this view, multiple endopeptidases^48–50^ and a dipeptidyl aminopeptidase^51^ are localized to the DV. Meanwhile, essential exopeptidases are primarily found in the cytoplasm, which are thought to further process these peptides into amino acids after they are transported out of the DV^6,52–54^. Therefore, compensating for the PfCRT functional defect by transporting amino acids directly is less likely. Our metabolomics analyses also indicate that peptide accumulation is the primary consequence of PfAAT1 knockdown. Consistent with this observation, disruption of *pbaat1* in *P. berghei* also preferentially affects Hb-derived peptides^23^.

**Figure 7:**
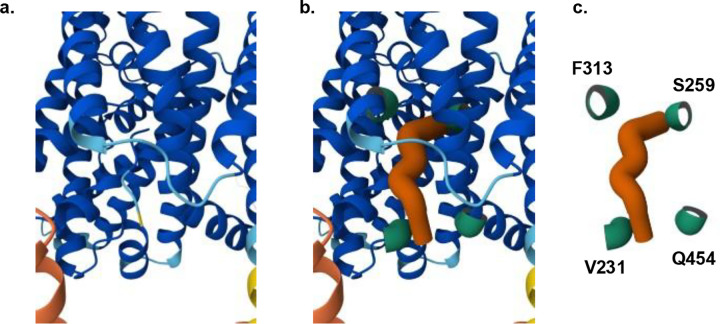
AlphaFold3 prediction of PfAAT1 in complex with the Hb-derived hexapeptide VDPV F. a, Cartoon representation showing a zoomed-in view of the hexapeptide within the putative peptide channel of PfAAT1. Colors indicate pLDDT confidence scores (>90, blue; 70–90, light blue; 50–70, yellow; <50, red). Model confidence metrics are ipTM = 0.93 and pTM = 0.69. b, The hexapeptide (brown) and four of the proximal residues (green) are highlighted in the same orientation as in a. c, Isolated view of the hexapeptide and the highlighted PfAAT1 residues.

AlphaFold 3^55^ prediction of PfAAT1 structure in complex with the Hb-derived hexapeptide VDPVNF places the peptide within a central channel of the protein, with an interface predicted TM-score (ipTM) of 0.93, indicating high confidence in the relative positioning of the peptide and protein (Fig. 7 and **Supplementary Fig. 12**). PfAAT1 residues within 5 Å of the hexapeptide include F313, V231, and Q454, as well as S259, which lies adjacent to the field-mutated residue S258. Although interpretation of this model requires caution, it further supports the possibility of Hb-derived peptide transport by PfAAT1 suggested by our results and implicates field-mutated residues in shaping peptide transport specificity and efficiency.^51^

While our data support peptide transport as the primary function of PfAAT1, we have not fully excluded the possibility that PfAAT1 contributes directly to amino acid transport, as detection of disrupted amino acid flux from the DV to the cytoplasm may be limited by the experimental design. In contrast to Hb-derived peptides, amino acids are distributed across multiple cellular compartments. As metabolite levels were measured at the whole-cell level rather than within the DV, their accumulation in the DV upon PfAAT1 knockdown may go unnoticed. Notably, cysteine and methionine, two amino acids identified in our functional assays as PfAAT1-dependent, are among the least abundant amino acids in human hemoglobin (6 each). Another rare amino acid in hemoglobin is tryptophan, which was previously found to be a potential substrate of PfAAT1^22^. Their low abundance may further limit the sensitivity of metabolomic detection.

A potential caveat of the amino acid–limitation experiments is that depletion of a specific amino acid from the culture medium would be expected to increase aTc EC_50_ only if the parasite normally obtains a substantial fraction of that amino acid from the medium and reduced extracellular availability increases reliance on PfAAT1-dependent transport. Methionine is a case in point. It was previously demonstrated that *P. falciparum* acquires methionine from the extracellular environment. At the same time, a *pfcrt* L272F mutation that causes swollen DVs and accumulation of Hb-derived peptides, phenotypes consistent with impaired peptide transport from the DV, also rendered parasites methionine auxotrophs. Together, these findings suggest that methionine is normally obtained from both the extracellular environment and Hb digestion within the DV, allowing defects in one source to be compensated by the other^56,57^. Conversely, if an amino acid is primarily obtained from Hb digestion, removing it from the medium may not alter aTc EC_50_, even if PfAAT1 participates in transporting that amino acid or related peptides. Similarly, interpretation of comparative growth between parasite lines expressing distinct *pfaat1* alleles is constrained by the same consideration. Differences in growth under a given amino acid–limited condition suggest allele-dependent differences in its acquisition, whereas the absence of a difference does not exclude that amino acid as a PfAAT1 substrate. Therefore, these assays do not comprehensively define the substrate range of PfAAT1 or fully resolve functional differences between alleles.

We also note that the observation that WT *pfcrt* fully rescues the PfAAT1 knockdown phenotype in Dd2 (Fig. 2a), whereas PfAAT1 knockdown still produces a mild growth defect in NF54 (Fig. 1d), suggests that additional genetic factors in the Dd2 background may contribute to compensating for PfCRT dysfunction. Identifying these factors will be important for fully understanding the adaptive landscape of CQ resistance^58^.

PfCRT mutations have been implicated not only in CQ resistance but also in altered susceptibility to multiple antimalarial drugs^59^, potentially contributing to the persistence of mutant pfaat1 alleles. Notably, WT *pfcrt* alleles have re-emerged in the field following withdrawal of CQ treatment^60–62^, whereas mutant *pfaat1* alleles remain highly prevalent^63^. Several explanations remain possible for this observation. Although our data show that mutant PfAAT1 variants confer a fitness advantage in a mutant *pfcrt* background, these variants may also provide a more general gain-of-function effect independent of *pfcrt* genotype. Alternatively, *pfaat1* mutations may remain under positive selection through direct effects on antimalarial susceptibility or indirect compensation for other resistance-associated fitness costs. For example, activation of artemisinins, the current frontline antimalarials, depends on heme released during Hb digestion^64–66^, and artemisinin resistance has been associated with reduced Hb endocytosis and Hb digestion^67^. Under such conditions, enhanced transport of Hb-derived peptides or amino acids could become advantageous and favor PfAAT1 variants with increased transport activity. Future studies examining the effects of *pfaat1* alleles across diverse drug-resistant genetic backgrounds will therefore be important for understanding the broader evolutionary significance of these mutations.

Overall, our findings highlight the critical dependence of *P. falciparum* on Hb-derived nutrients and underscore peptide transport pathways as key nodes of vulnerability that may be exploited for antimalarial drug development.

## Methods:

### Maintenance of parasite cultures

*P. falciparum* parasites without selectable markers were cultured in RPMI 1640 medium (Gibco) supplemented with 0.25% (wt/vol) Albumax (Gibco), 15 mg/L hypoxanthine, 110 mg/L sodium pyruvate, 1.19 g/L HEPES, 2.52 g/L sodium bicarbonate, 2 g/L glucose and 10 mg/L gentamicin. Hematocrit was maintained at 2%. Endogenously tagged PfAAT1 conditional knockdown lines were maintained in blasticidin S (2.5 μg/ml; Thermo Fisher Scientific) and 500 nM aTc (Sigma-Aldrich, 37919). DSM-1 (2 μM; Asinex) was added to parasite cultures expressing PfAAT1 constructs from the *cg6* locus. Deidentified human RBCs were obtained from the St. Louis Children’s Hospital blood bank.

Parasite cultures were maintained in a synchronized state. Briefly, asynchronous parasite cultures were washed with RPMI medium and passed through a MACS LD magnetic column (Miltenyi Biotec). Older parasites (>28 h post invasion) were retained on the column because of the presence of paramagnetic hemozoin crystals. Parasites were eluted into prewarmed culture medium at 2% hematocrit and incubated for 3 h to allow egress and invasion. Cultures were then treated with 5% sorbitol at 37 °C for 10 min to osmotically lyse older parasites, leaving newly invaded ring-stage parasites intact^68^. Synchronized parasites were subsequently maintained below 5% parasitemia with continuous shaking at 90 r.p.m. Cultures were maintained at 37 °C in chambers containing 5% O2, 5% CO2 and 90% N2.

For experiments using amino acid–depleted media, base medium was prepared using RPMI 1640 medium without amino acids or sodium phosphate (US Biological, R8999–04A) supplemented with 0.25% (wt/vol) Albumax (Gibco), 15 mg/L hypoxanthine, 110 mg/L sodium pyruvate, 7.19 g/L HEPES, 2.52 g/L sodium bicarbonate, 2 g/L glucose, 0.8 g/L anhydrous sodium phosphate (dibasic), 300 mg/L glutamine, 50 mg/L isoleucine and 10 mg/L gentamicin. All remaining amino acids (Sigma) were individually added to match the concentrations present in standard RPMI 1640 medium (Gibco) to generate complete medium. Specific amino acid–depleted media or media lacking defined groups of amino acids were prepared by omitting the indicated amino acids.

### Plasmid construction and transfection

To endogenously tag *pfaat1* (PF3D7_0629500) using CRISPR–Cas9 genome editing, a gRNA targeting the region 12–31 bp upstream of the stop codon was cloned into the AflII site of the pAIO3 vector^69^, which encodes SpCas9 and the *P. falciparum* U6 regulatory element. A linear vector pSN054 was used as the backbone for the repair templates^70^. A 591 bp genomic region immediately upstream of the *pfaat1* stop codon was used as the left homology region and cloned into the FseI–AsiSI sites. Two synonymous mutations were introduced within the gRNA-targeted region to prevent Cas9 re-cleavage following genomic integration. A 627 bp genomic region downstream of the stop codon was used as the right homology region and cloned into the I-SceI site.

For allelic replacement of PfAAT1^F313S^ with PfAAT1^WT^, a 522 bp genomic region ending immediately upstream of codon 312 was used as the left homology region. Codons encoding amino acids 312–606 were recodonized using human codon bias (IDT) and inserted immediately downstream of the left homology region.

To complement PfAAT1 conditional knockdown parasites, the coding sequence of PfAAT1 was amplified from NF54 mRNA isolated using TRIzol (Thermo Fisher Scientific) and the SuperScript RT–PCR kit (Invitrogen). Amplified products were cloned into the XhoI–NotI sites of the p-y-EOE-attP vector using In-Fusion cloning. N-terminal V5 tags were introduced through the amplification primers. Specific mutations were introduced using the QuikChange Lightning Multi Site-Directed Mutagenesis kit (Agilent Technologies).

pSN054-based plasmids were electroporated into BigEasy electrocompetent *E. coli* (Lucigen), whereas all other plasmids were transformed into TOP10 Ultracompetent *E. coli* (Invitrogen). Plasmids were isolated using the NucleoBond Xtra Midi kit (Macherey–Nagel) and sequence-verified by Plasmidsaurus before parasite transfection, as previously described^71^. Modified pAIO3 and pSN054 plasmids were co-transfected into parasites, which were subsequently selected with blasticidin S and aTc. Proper integration at the *pfaat1* locus was verified by diagnostic PCR using primers positioned outside the homology regions, followed by sequence verification of the PCR products by Plasmidsaurus.

Complementation plasmids containing an attP site were integrated into the *cg6 attB* locus of NF54 and Dd2 parasites by co-transfection with the pINT plasmid encoding Bxb1 integrase^26^. Recombinant parasites were selected with DSM-1 in addition to blasticidin S and aTc.

All primers used for cloning and sequencing were purchased from IDT and are listed in Supplementary Table S1.

### aTc washout and readdition

Synchronized parasite cultures at the schizont-to-ring transition stage were washed five times with complete RPMI medium, with the first wash performed in 5% sorbitol for 10 min at 37 °C to obtain synchronized ring-stage parasites at the beginning of each experiment. Cultures were then divided into multiple aliquots depending on the experimental design. Unless otherwise specified, aTc was added to +aTc cultures at a final concentration of 500 nM, whereas an equal volume of DMSO was added to the −aTc cultures.

### Preparation of protein samples, SDS–PAGE and western blotting

Parasites from 12–36 ml cultures were first treated with 0.035% saponin in 1× PBS for 5 min on ice to lyse erythrocytes. Parasite pellets were then resuspended in lysis buffer (150 mM NaCl, 50 mM Tris-HCl, pH 7.5, and 1% NP-40 supplemented with protease inhibitor cocktail) and incubated on ice for at least 10 min with occasional mixing. Lysates were clarified by centrifugation and the supernatants were collected. 4X sample buffer supplemented with β-mercaptoethanol was added to the lysates before SDS–PAGE analysis.

Fifteen microliters of each sample were resolved on 4–15% gradient SDS–PAGE gels (Bio-Rad) and transferred to PVDF membranes for western blotting. Primary antibodies used were mouse anti-HA (Roche Clone 3F10; 1:1,000), mouse anti-V5 (Thermo fisher R960; 1:1,000), rabbit anti-HA (Sigma H6908; 1:1,000), rabbit anti-V5 (Invitrogen PA1–993; 1:1,000), mouse anti-GFP (Takara clone JL8; 1;1000), and mouse anti-PM V^48^ (1:250). IRDye-conjugated goat secondary antibodies (LI-COR) were used at 1:15,000 dilution. Membranes were incubated with primary antibodies overnight at 4 °C and with secondary antibodies for 1 h at room temperature. LI-COR Odyssey blocking buffer was used for blocking and primary antibody dilution. PBS supplemented with 0.1% Tween-20 was used for secondary antibody dilution and all washing steps. Blots were imaged using a LI-COR Odyssey imaging system and images were processed using Image Studio Lite 5.2 (LI-COR).

### Microscopy

Thin smears of parasite-infected RBCs were stained using Harleco Hemacolor stains (catalog no.: 65044) to assess digestive vacuole morphology. Images were acquired using a Zeiss Axio Observer.D1 microscope at the Washington University Molecular Microbiology Imaging Facility.

For immunofluorescence microscopy, trophozoite-stage parasites were incubated on concanavalin A–coated glass coverslips for 30 min before fixation with 4% paraformaldehyde and 0.075% glutaraldehyde. Parasites were permeabilized with 0.05% Triton X-100. Blocking and antibody dilutions were performed in 3% BSA, and washes were carried out using 1× PBS. Mouse anti-V5 (Invitrogen PA1–993; 1:500) and rabbit anti-HA (Sigma H6908; 1:100) antibodies were used as primary antibodies. Goat anti-mouse Alexa Fluor 488 and goat anti-rabbit Alexa Fluor 555 secondary antibodies (Invitrogen; 1:2,000) were used for detection. Coverslips were mounted using ProLong Gold Antifade reagent with DAPI (Thermo Fisher Scientific), cured for 24 h and imaged using a Zeiss AxioImager.M1 epifluorescence microscope equipped with a Hamamatsu ORCA-ER CCD camera and AxioVision v.4.8.1 software.

For immuno-electron microscopy infected RBCs were fixed in 4% paraformaldehyde in 100 mM PIPES/0.5 mM MgCl2 (pH 7.2) for 1 h at 4 °C, embedded in 10% gelatin, and infiltrated overnight with 2.3 M sucrose/20% polyvinyl pyrrolidone. Samples were frozen in liquid nitrogen, sectioned (50 nm) using a Leica Ultracut UCT cryo ultramicrotome, and blocked with 5% FBS/5% NGS. Sections were incubated with Anti-GFP (Invitrogen; 1:200) followed by 12 nm colloidal gold-conjugated secondary antibody (1:30). Sections were stained with 0.3% uranyl acetate/2% methyl cellulose and imaged using a JEOL 1200EX transmission electron microscope equipped with an AMT 8 megapixel digital camera.

### Comparative growth experiments

Parasite growth curves were determined using at least two technical replicates for each +aTc and −aTc condition in 1 ml cultures in 24-well plates, seeded at an initial parasitemia of 1% ring-stage parasites. Parasitemia was measured every 24 h by flow cytometry using a BD FACSCanto system following staining with acridine orange (1.5 μg/ml in PBS). Parasites were subcultured as needed to prevent overgrowth. Parasitemia values were quantified using BD FACSDiva software. Uninfected RBC controls were included in each plate to determine the background signal.

Comparative growth between PfAAT1 conditional knockdown and WT conditions was assessed by calculating the area under the curve (AUC) from cumulative parasitemia-versus-time curves using GraphPad Prism, with Y = 0 used as the baseline. Because +aTc and −aTc cultures were generated as aliquots from the same synchronized culture, no normalization to the initial parasitemia was performed. Relative growth for each biological replicate was calculated as the ratio of the AUC of the −aTc culture to that of the +aTc culture. Ratios were multiplied by 100 and reported as percentage growth.

Comparative growth of PfAAT1 allelic replacement strain was determined under +aTc conditions using the same experimental strategy, except that parasites were washed using RPMI medium supplemented only with glutamine and isoleucine before media replacement. To account for variation in initial parasitemia between independently cultured parasite lines, parasitemia values were normalized to the mean Day 1 parasitemia for each strain within each biological replicate.

AUC values for parasites expressing PfAAT1^WT^ were normalized to those of parasites expressing PfAAT1^F313S^ under the same media condition within each biological replicate. Normalized ratios were multiplied by 100 and reported as percentage fitness.

Unpaired Student’s t-tests were used when comparing two groups. For comparisons involving more than two groups, ordinary one-way ANOVA followed by Bonferroni’s multiple-comparison test was performed.

### Metabolomics of PfAAT1 conditional knockdown lines:

*Mycoplasma* contamination was assessed before sample collection using e-Myco Mycoplasma detection PCR kit (BullDog-Bio). *Mycoplasma*-free parasites were sorbitol-synchronized for at least two generations. ATc washout was performed in the ring stage of the experimental cycle and 30-hour post-invasion trophozoites were magnetically enriched using MACS CS columns on a SuperMACS^™^ II Separator (Miltenyi Biotec) to remove uninfected red blood cells. Trophozoite counts were determined by flow cytometer (iQue^®^3, Sartorius). 10^8^ parasites were lysed in 1 mL of 90% cold methanol containing 0.5 μM [^13^C_4_,^15^N_1_]-aspartate (Cambridge Isotope Laboratories) as an internal standard. Lysates were vortexed to disrupt pellets, centrifuged at 13,000 × g for 10 min, and supernatants were harvested. Any residual insoluble cell pellet was removed by centrifugation (15000 rpm and 4°C for 20 minutes), the 90% MeOH supernatant was transferred to a new Eppendorf Tube and dried down under nitrogen gas flow. Samples were then resuspended in HPLC-grade water spiked with 1 μM chlorpropamide (Alfa Aesar) as an internal standard to account for LC-MS instrument signal detection variability. A pooled quality control sample was prepared by aliquoting equal volumes of each sample together which were assayed periodically along with blank samples throughout the randomized LC-MS run to ensure consistency in signal detection across the measurement period.

Two microliters of each sample were injected into a Thermo Scientific Dionex Ultimate 3000 liquid chromatography system, and a reverse phase method was used for separation of metabolites with a XSelect HSS T3 2.5μM C18 column (Waters, 186006151). Mobile phase A was 3% Methanol supplemented with 10mM tributylamine, 15mM acetic acid and 2.5μ medronic acid, and Mobile phase B was 100% methanol. The mobile phase gradient for the 25-minute method is as follows with the percentage being the amount of mobile phase B: 0% from 0–5.0 minutes, 20% from 5.0–13.0 minutes, 55% from 13.0–15.0 minutes, 65% from 15.0–17.5 minutes, 95% from 17.5–21.0 minutes and 0% from 21.0–25.0 minutes. The eluate was then sent into a Thermo Scientific Q Exactive Plus Orbitrap mass spectrometer. Ion detection was run in negative mode with a scan range of 85–1000 m/z with a resolution of 140,000 at an m/z of 200.

Following data collection, the .raw files were converted to .mzML using MSConvert^72^ with the parameters set to peakPicking with vendor msLevel 1. Files were uploaded to ElMaven^73^ for alignment and peak calling by matching m/z and retention time to previously run metabolite standards. Following analysis, the areas of each called peak were exported and the chlorpropamide area was used as an internal standard to correct for instrument variability. The blanks average was subtracted from the sample values followed by the calculation of relative standard deviation (RSD) of the QC injections. Any metabolite that had an RSD >30 was discarded from further analysis.

For each technical replicate, metabolite counts were normalized to the median metabolite abundance of that sample. Average abundance values for each metabolite were then calculated across technical replicates and log2-transformed before downstream analyses. Volcano plots were generated using multiple paired t-tests, and false discovery rate (FDR) correction for multiple comparisons was performed using the two-stage step-up method of Benjamini, Krieger and Yekutieli. All analyses were performed using GraphPad Prism version 11.0.

Three separate paired analyses were performed using the same statistical approach: NF54 (+aTc versus −aTc; n = 3 biological replicates), Dd2 (+aTc versus −aTc; n = 3 biological replicates) and a combined analysis including both strains (n = 6 biological replicates).

For metabolite class analyses, metabolites were grouped into three categories: Hb-derived peptides, amino acids and all other detected metabolites. Log_2_ fold changes were compared between groups using a non-parametric Kruskal–Wallis test followed by Dunn’s multiple-comparison test. Individual amino acid and Hb-derived peptide abundances were compared using paired t-tests.

### aTc EC_50_ determination in different media

Ring-stage parasites were washed five times with medium containing only glutamine and isoleucine (+QI) and maintained for 2 days in the presence of aTc to adapt parasites to amino acid–deprived conditions. After 2 days, synchronized schizont-to-ring stage cultures were subjected to aTc washout. Parasites were then seeded into 96-well plates at 0.4% parasitemia and 2% hematocrit in 180 μl of the indicated media conditions. Twenty microliters of serially diluted aTc prepared in +QI medium were added to each well. Parasites were incubated for 120 h before staining with PicoGreen.

EC_50_ values were determined from normalized dose–response curves using GraphPad Prism v11. Within each biological replicate, EC_50_ values obtained under each amino acid–depleted condition were normalized to the EC_50_ determined in complete medium, in which all amino acids were individually supplemented to standard RPMI 1640 concentrations. Statistical comparisons were performed using ordinary one-way ANOVA followed by Bonferroni’s multiple-comparison test, comparing each amino acid–depleted condition with the complete medium control.

### Analysis of *pfcrt*–*pfaat1* allele co-occurrence

Sample-level summaries of PfAAT1 and PfCRT alleles were downloaded from the PfHaploAtlas database^34^ and matched using sample identifiers. Samples containing heterozygous calls, missing genotypes or low-coverage calls for either gene were excluded from the analysis. PfCRT alleles were grouped according to the amino acid present at position 76 (K76 or T76), irrespective of other mutations in the gene. PfAAT1 alleles were grouped into three categories: WT alleles without mutations, alleles containing either or both S258L and F313S mutations, and all other alleles. Chi-square analysis was performed to assess allele co-occurrence. In Fig. 4d, the V231D PfAAT1 allele is displayed separately from the remaining “other” alleles.

### Chloroquine dose–response assays

Synchronized ring-stage parasite cultures at 0.2% parasitemia were seeded into 96-well plates following aTc washout and readdition. Serially diluted CQ prepared in complete medium was added to the cultures, and parasite growth was quantified after 72 h using PicoGreen staining. IC_50_ values were determined from normalized dose–response curves using GraphPad Prism. Student’s t-tests were used for comparisons between two groups, whereas ordinary one-way ANOVA followed by Bonferroni’s multiple-comparison test was used for comparisons involving more than two groups.

### Alphafold predictions

Wild-type PfAAT1 sequence and hexapeptide VDPVNF were used as protein inputs and sodium as an ion input in Alphafold 3 server. Residues in the hexapeptide were selected as a target to identify PfAAT1 residues within 5 Å of the hexapeptide.

## Supplementary Material

Supplementary Files

This is a list of supplementary files associated with this preprint. Click to download.

- supfile1.xlsx
- Supplementaryfiguresandtable.pdf
- supfile2.xlsx
